# Exploring the effectiveness of eHealth interventions in treating Post Intensive Care Syndrome (PICS) outcomes: a systematic review

**DOI:** 10.1186/s13054-024-05089-6

**Published:** 2024-09-27

**Authors:** Daniel Jie Lai, Zhao Liu, Elaine Johnston, Lisa Dikomitis, Teresa D’Oliveira, Sukhi Shergill

**Affiliations:** 1 Canterbury Christ Church University, Canterbury, UK; 2 National Institute of Health and Care Research, Applied Research Collaboration Kent, Surrey and Sussex, UK; 3 School of Computing, University of Kent, Canterbury, UK; 4 College of Software and Big Data, Inner Mongolia Electronic Information Technical College, Hohhot, Inner Mongolia China; 5 Maidstone and Tunbridge Wells NHS Trust, Kent, UK; 6 Kent and Medway Medical School, University of Kent and Canterbury Christ Church University, Canterbury, UK; 7 Centre for Health Services Studies, University of Kent, Canterbury, UK; 8 Institute of Psychiatry, Psychology, & Neuroscience, King’s College London, London, UK

**Keywords:** Critical care, Critical Illness, Critical care rehabilitation, Post-intensive care syndrome, EHealth, Digital health technologies

## Abstract

**Background:**

It remains unclear how to optimise critical care rehabilitation to reduce the constellation of long-term physical, psychological and cognitive impairments known as Post Intensive Care Syndrome (PICS). Possible reasons for poor recovery include access to care and delayed treatment. eHealth could potentially aid in increasing access and providing consistent care remotely. Our review aimed to evaluate the effectiveness of eHealth interventions on PICS outcomes.

**Methods:**

Studies reporting eHealth interventions targeting Post Intensive Care Syndrome outcomes, published in Medline, CINAHL, PsycINFO, Embase, and Scopus from 30th January 2010 to 12th February 2024, were included in the review. Study eligibility was assessed by two reviewers with any disagreements discussed between them or resolved by a third reviewer. Study quality and risk of bias were assessed using the Mixed Method Appraisal Tool. Further to the identification of effective strategies, our review also aimed to clarify the timeline of recovery considered and the outcomes or domains targeted by the interventions.

**Results:**

Thirteen studies were included in our review. Study duration, eHealth intervention delivery format, and outcome measures varied considerably. No studies reported a theory of behavioural change and only one study was co-produced with patients or carers. Most studies were conducted in the early post-discharge phase (i.e., < 3 months) and had feasibility as a primary outcome. The cognitive domain was the least targeted and no intervention targeted all three domains. Interventions targeting the psychological domain suggest generally positive effects. However, results were underpowered and preliminary. Though all studies were concluded to be feasible, most studies did not assess acceptability. In studies that did assess acceptability, the main facilitators of acceptability were usability and perceived usefulness, and the main barrier was sensitivity to mental health and cognitive issues.

**Conclusion:**

Our systematic review highlighted the promising contributions of eHealth with preliminary support for the feasibility of interventions in the early stages of post-critical care rehabilitation. Future research should focus on demonstrating effectiveness, acceptability, the cognitive domain, and multi-component interventions.

**Supplementary Information:**

The online version contains supplementary material available at 10.1186/s13054-024-05089-6.

## Background

Post Intensive Care Syndrome (PICS) has been increasingly recognised as an urgent problem among critical care survivors [1–4]. This is characterised as a sequalae of new or worsened physical, psychological, and cognitive impairments after critical illness which has significant impacts on functional outcomes, Health-Related Quality of Life (HRQoL), and employment [5–8]. The establishment of a rehabilitation pathway is essential for successful PICS management.

Critical care rehabilitation consists of four phases: acute recovery and prevention within the critical care unit, recovery in the hospital ward, the first 3 months after hospital discharge termed the early post-discharge period, and the late post-discharge period which can span years after discharge [9]. Our review terms the three phases after critical care discharge as the ‘post-critical care’ phases. The effectiveness of current interventions in the post-critical care phases are limited with most targeting the late post-discharge period [10, 11]. This limited effectiveness could be due to the time points chosen to begin rehabilitation (i.e., a later start of rehabilitation). The early post-discharge period is deemed a crucial recovery point where critical care survivors are most vulnerable. These impacts are further magnified by regional health inequalities that restrict access to care [12]. There is a need for earlier intervention and continuity of care.

The use of electronic Health (eHealth) is presented by the literature as a solution to minimise health inequalities and facilitate earlier intervention. eHealth technologies are characterised by 1) enabling the storage, retrieval, and transmission of data, 2) supporting clinical decision-making, and 3) facilitating remote care [13]. These technologies include mobile applications, video conferencing, virtual reality, web platforms and wearable technology. The use of eHealth has proliferated within critical care. For example, the tele-critical care model aids in addressing workforce shortages, provides better access to specialist expertise, reduces patient transfers, and lowers ICU mortality [14, 15]. However, efforts in harnessing the benefits of eHealth have only just begun in post-critical care.

Studies conducted in the last 3 years demonstrate a demand for tools that can detect and measure rehabilitation of PICS symptoms [16]. The use of eHealth interventions to rehabilitate patients in the early post-discharge phase could promote better PICS recovery. Evidence from other chronic patient populations like heart failure, stroke and diabetes has shown promising results in eHealth’s effectiveness on post-hospital disease management, medicine adherence, and health-related quality of life [17–19]. However, specific identification and evaluation in a post-critical care setting has yet to be done. To our knowledge, this is the first comprehensive review of eHealth’s impact on PICS outcomes during the critical care rehabilitation phase. This encompasses the in-hospital, early, and late post-discharge phases. The objective of the review is to identify effective strategies using eHealth that target PICS, their timeline in the recovery path and the outcomes addressed. As primary outcomes, we consider the PICS domains (physical, psychological and cognitive) targeted by the eHealth interventions, the recovery phase these interventions are implemented and their effectiveness. Secondary objectives include the feasibility of these eHealth interventions, acceptability, and identification of the barriers, and facilitators of eHealth intervention uptake.

## Methods

This systematic review is reported based on the Preferred Reporting Items for Systematic Reviews and Meta-Analyses (PRISMA) [20]. The study was registered and published in the International Prospective Register of Systematic Review databases (PROSPERO registration number: CRD42023463036) [21]

### Search strategy and selection criteria

#### Search strategy

The following databases were searched: Medline, CINAHL, PsycINFO, Embase, and Scopus. Reference lists from key articles were also checked for any additional articles that fit the inclusion criteria. Due to the rapid innovation of eHealth technologies, studies that were published from 30th January 2010 to 12th February 2024 were included in this review. No restrictions were imposed on the language of publication.

The PICO framework [22] was used to identify key terms and develop the search string. PIO was used as there was no restriction imposed on the study design. The comparator category was not included in the search strategy to expand the articles picked up. The categories were defined as (P): Post Intensive Care patients; (I): eHealth interventions; (O) Post Intensive Care Syndrome outcomes (Physical, Psychological, Cognitive). The search string was tailored to fit the querying format of each database and can be found in Supplementary Material S1.

#### Study inclusion and exclusion criteria

Eligible studies included i) adults over the age of 18 who have been discharged from critical care (in the hospital ward, early post-discharge, and late post-discharge), ii) the inclusion of one or more eHealth interventions implemented in any of the three phases of post-critical care recovery, iii) PICS domains were measured as an outcome, vi) full text published in peer reviewed journals. There were no restrictions made on the study design and the language of publications. As current eHealth definitions proposed in the literature are very broad and general, we operationalised what constitutes an eHealth intervention using the definition by Black et al., [13] which was conceptualised to aid the categorisation of eHealth interventions using themes generated from 53 systematic reviews. The eHealth inclusion and exclusion criteria were developed based on this definition and the types of eHealth interventions were categorised in these categories.

1. Telemedicine
2. Telerehabilitaiton
3. Self-directed interventions
4. Remote patient monitoring (wearables, sensors)
5. Virtual Reality (VR)

Studies excluded consisted of (i) no evidence of eHealth intervention, (ii) Paediatric (children) ICU, (iii) neonatal/prenatal ICU, (iv) systematic reviews and meta-analyses, (v) conference abstracts, and (vi) study protocols.

#### Selection process

Two reviewers (DL, ZL) independently screened the articles according to the stipulated inclusion and exclusion criteria. During the titles and abstract screening stage, screening procedures proposed by Adams et al. [23] were used. The first reviewer (DL) screened all titles and abstracts, while the second reviewer (ZL) screened a 10% random selection of articles. There was substantial inter-rater reliability between the reviewers (Kappa = 0.66; percentage agreement = 98.8%). Full-text screening was done independently by DL and ZL with almost perfect agreement (Kappa = 0.95, percentage agreement = 98.3%) Any disagreements were discussed between the two reviewers until a consensus was reached. When consensus could not be reached, the dispute was solved with the consultation of a senior team member (TD).

### Data extraction

Data extracted consisted of study characteristics (Author/year; Country; Study design; Population; Post-critical care timepoint; Sample size/Control (if any); Study duration), eHealth intervention characteristics (Intervention; Type of eHealth intervention; Delivery Format; Outcome Measures; Findings).

Feasibility was measured and assessed in different ways due to the variation of eHealth interventions. Feasibility data extracted included the feasibility outcome defined by authors, attrition, definition of intervention adherence, adherence rate, reasons for participant withdrawal, and author’s conclusions.

Data extracted for acceptability consisted of how acceptability was assessed (acceptability measure), main findings, and reported barriers and facilitators in intervention uptake. Data extraction was done in duplicate by two reviewers (DL and ZL) who worked independently.

#### Risk of *bias* and quality assessment

Two reviewers (DL, and ZL) independently assessed the risk of bias and the quality of studies using the Mixed Methods Appraisal Tool (MMAT) [24]. The tool has 5 quality criteria examining and evaluating the appropriateness of a study’s aims, methodology, design, data collection, data analysis, presentation of findings, discussion, and conclusion. The quality criteria are rated with ‘Yes’, ‘No’, or ‘Can’t tell’ and are evaluated based on study design. Criteria for a randomised controlled trial are different from a non-randomised trial (quality criteria can be found in Supplementary Material S2). Each study was scored using percentages based on the recommendations by Pace et al. [25]_._ Any disagreements were resolved through discussion between the two reviewers.

### Data synthesis and analysis

A quantitative analysis of outcomes or meta-analysis could not be done due to the heterogeneity of the study designs, outcome measures used, eHealth interventions, and the critical care population. With the included studies, a qualitative narrative synthesis was undertaken to summarise the primary and secondary outcomes of interest. Data were grouped based on the main outcomes listed in the data extraction section.

## Results

**Fig. 1:**
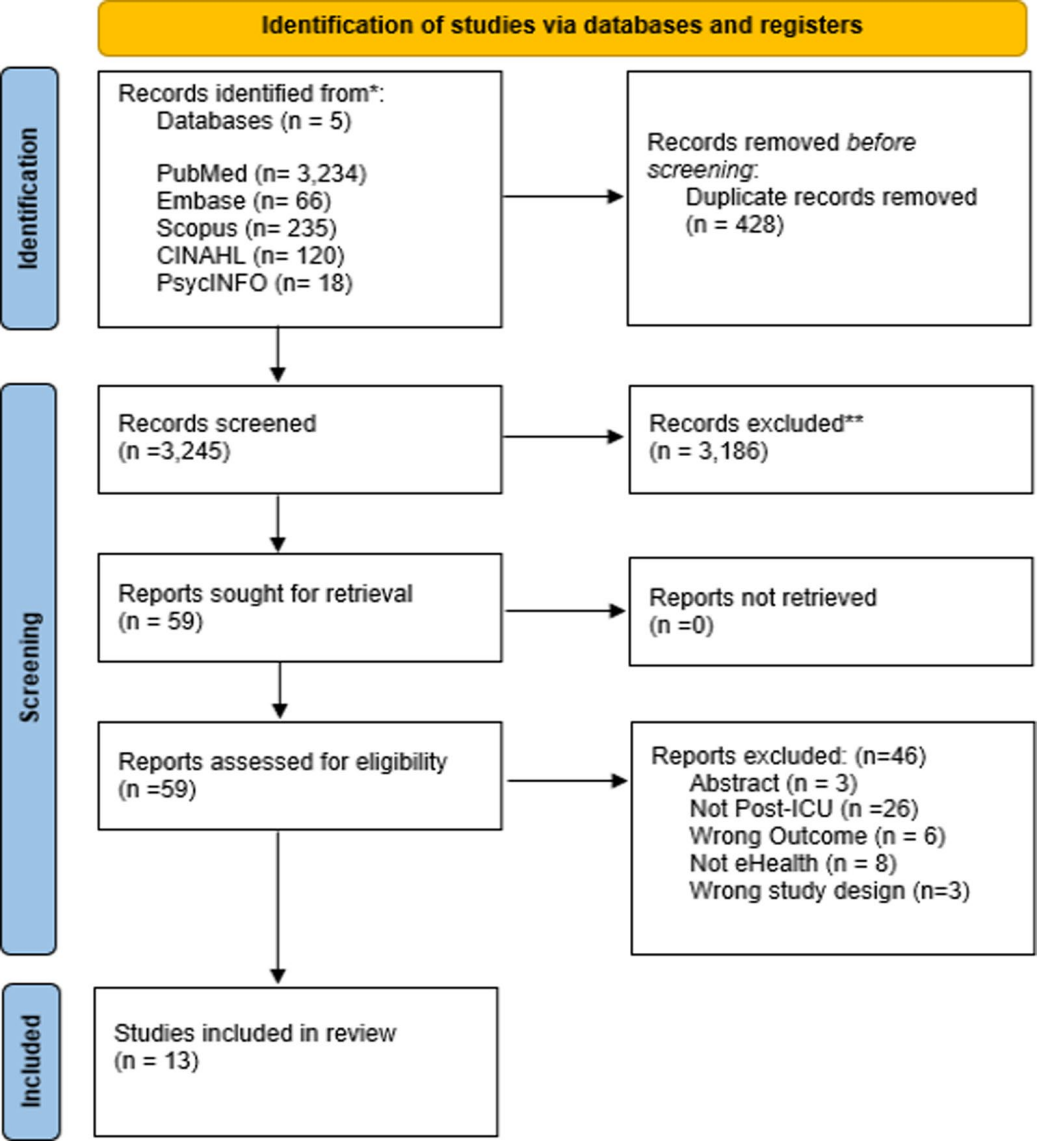
PRISMA (Preferred Reporting Items for Systematic Reviews and Meta-Analyses) flow diagram documenting the processes of identification, screening, and article inclusion. Latest search 12th February 2024

Initial database searches yielded 3,673 articles. The deduplication of 428 articles led to a total of 3,245 titles and abstracts screened. In accordance with the exclusion criteria, 3,186 articles were excluded leaving 59 articles for full-text retrieval. Out of the 59 articles, 13 met the inclusion criteria for the current review. Figure 1 presents the PRISMA diagram documenting the processes of identifying, screening, and selecting included papers.

### Study characteristics

Studies were conducted across 7 countries with the majority coming from the United States (6/13). A total of 548 participants were enrolled across 13 studies. The sample sizes ranged from 5 to 89 with participant ages ranging from 47 to 72 years. Study design varied considerably across the studies with 46% (6/13) of studies being Randomised Controlled feasibility Trials (RCT) [31–33, 36, 41, 42], 38.4% (5/13) prospective observational cohort studies [34, 35, 38, 40, 43], and 15.3% (2/13) qualitative studies [37, 39]. None of the studies reported any underpinning theory of behaviour change and only 1 study [40] reported co-production efforts during intervention development. The characteristics and intervention descriptions of included studies are summarised in Table 1.

**Table 1:** Study characteristics, description of eHealth interventions and main findings

Author, Year	Country	Study Design	Population	Time point	Sample/Control	Duration	Intervention	Type of eHealth intervention	Delivery format	Outcome Measures	Main Findings
Balakrishnan et al. [26]	US	Feasibility RCT	Covid-19 patients	Early post-discharge	40/TAU^§^	10 weeks	Telemedicine visits 14 days post-hospital discharge in Covid-19 and ARDS survivors	Telemedicine/ Video call	Video call	6MWT EQ5D5L	No significant difference in HRQOL, and anxiety
Capin et al. [27]	US	Feasibility RCT	Covid-19 patients	Early post-discharge	44/Active^*^	12 weeks	Telerehabilitation program which included 12 exercise sessions led by a physiotherapist	Tele-rehabilitation	App	30 s Chair stand test TUG Step count MoCA	No significant difference in physical outcomes No significant difference in cognitive outcomes
Cox et al. [28]	US	Feasibility RCT	Medical, cardiac, and surgical ICU	Early post-discharge	80/Active^*^	12 weeks	Self-directed mindfulness application which delivered 4 mindfulness sessions consisting of background videos, guided meditation and educational materials	Self-Directed eHealth intervention	App	PHQ-15 PTSS PHQ-9 GAD-7 EQ5D5L	Mindfulness group has lower PTSD and Depressive symptoms and anxiety scores compared to group that received education Feasibility, acceptability, and usability concluded
Denehy et al. [29]	Australia	Observational Quasi-experimental	Medical ICU	In-hospital	53	1 week	Critical care survivors wore an actigraph for 7 days at 2 months post-hospital discharge	Remote patient monitoring	Wearable	6MWT TUG SF-36 Step count	Absence of chronic disease was significantly associated with increased distance walked (p < .000) where chronic disease explained 33.5% of the variance in average distance walked ICU survivors were inactive when quantitatively measured at 2 months after hospital discharge 63% of participants did not reach 30 min of moderate physical activity
Estrup et al. [30]	Denmark	Observatinal Quasi-experimental	Medical, surgical ICU	In-hospital	44	1 week	Activity levels measured for 7 days in-hospital. Physical function outcomes were measured 3 months post-hospital discharge	Continuous patient monitoring	Wearable	CPAx Step count	Improved physical function in most patients 3-month post-hospital discharge Physical function correlated with mean daily activity (p = 0.017), maximum activity on second day (p = .0053), and total activity in the daytime (p = 0.0058)
Jackson et al. [31]	US	Feasibility RCT	Medical, surgical ICU	Early post-discharge	21/TAU^§^	12 weeks	A hybrid cognitive, physical and functional rehabilitation. physical and functional rehabilitation sessions were conducted via video call. Cognitive rehabilitation was done in-person	Tele-rehabilitation	Video call	TUG Tower test MMSE	Improvements in cognitive outcomes Improvements in instrumental activities of daily living (driving, shopping)
Kovaleva et al. [32]	US	Qualitative	ICU Sepsis and/or ARDS diagnosis	Late post-discharge	24	12 weeks	Video conference at 3- and 12- weeks post hospital discharge	Telemedicine	Video call	Patient experience	All participants found telemedicine visit acceptable Technology was easy to use
Park et al. [33]	US	Observational Quasi-experimental	Covid-19	Late post-discharge	18	Up to 14 sessions	Up to 14 virtual, evidence-based psychotherapy sessions for patients. Intervention integrated psychoeducation, cognitive restructuring, acceptance, exposure-based, and mindfulness	Tele-psychotherapy	Video call	PHQ-9 GAD-7	Significant decrease in participants meeting anxiety (57.1% decrease) and depression (57.2% decrease)
Parker et al. [34]	Australia	Qualitative	ARDS survivors	Late post-discharge	10	N. A	Application which consisted of educational resources on rehabilitation, a mood tracker, and a goal tracker	Educational	App	Acceptability	80% stated application was easy to use 37% stated it would be helpful to have an application demonstration Median score for app ‘helpfulness’ was the maximum score of 5, IQR [4.24–5.00]
Rose et al. [35]^**¶**^	UK	Observational Quasi-experimental	Medical, Surgical ICU	Early post-discharge	5	12 weeks	Delivered through online/mobile platform. The platform provided assessments of baseline status and recovery barriers, tailored e-resources based on recovery barriers, patient recovery e-diary	Telemedicine	Web-based/App	Short-term goal completion	Two patients reported achieving short-term goals, 2 partially achieved goals, and 1 did not achieve
Vlake et al. [36]	Netherlands	Multi-centre feasibility RCT	ICU Sepsis or septic shock diagnosis	In-hospital	50/Active^*^	1 session	Patients in the stepdown ward go through the ICU-VR intervention which includes different scenes in the ICU unit. Patients were then followed up 2 days, 1 week, 1 month, and 6 months after VR intervention	Virtual Reality intervention	Virtual Reality headset	IES-R BDI-II MCS-12 EQ5D5L	PTSD and depression reduced in VR intervention group 2 days after exposure and persisted throughout follow up timepoints HRQoL improved in VR intervention group up to 1 month after VR intervention. Did not improve 6 months after intervention VR intervention group experienced greater sense of presence, involvement and experienced realism
Vlake et al. [37]	Netherlands	Multi-centre feasibility RCT	Covid-19	Late post-discharge	89/TAU^§^	1 session	VR intervention is the same as intervention above but was trialed on a post-hospital discharge sample. Participants were followed up 1 and 3 months after 3-month post-icu clinic appointment	Virtual Reality intervention	Virtual Reality headset	IES-R HADS SF-36 EQ5D5L	Psychological outcomes and quality of life did not improve Satisfaction in ICU aftercare rated higher in intervention group
Wood et al. [38]	Canada	Observational Quasi-experimental	Medical, Surgical ICU	Late post-discharge	70	2 sessions	A screening tool where participants completed 7 tasks within a VR environment	Virtual Reality Screening Tool	Virtual Reality	RBANS KINARM (VR Intervention)	Prevalence of cognitive and sensorimotor impairments 9/28 (32%) and 3/22 (14%) participants displayed global cognitive impairment Screening tool caused little to no procedural discomfort

None of the included studies reported an explicit behavioural theory or model of change

*RCT* Randomised Controlled Trial, *TAU* Treatment as usual, *HRQoL* Health Related Quality of Life, *PTSD* Post Traumatic Stress Disorder, *ICU* Intensive Care Unit, *M* Mean, *SD* Standard Deviation, *ARDS* Acute Respiratory Distress Syndrome, *VR* Virtual Reality

Outcome Measure Abbreviations: *6MWT* 6 Minute Walk Test*, EQ5D5L* 5 Europe Quality of Life 5 Dimensions 5 Levels, *TUG* Timed Up and Go Test, *MoCA* Montreal Cognitive Assessment, *PHQ-9/15* Patient Health Questionnaire (9 or 15 question version), *GAD-7* Generalised Anxiety Disorder – 7 questions, *PTSS* Post Traumatic Syndrome Scale, *SF-36* Short Form- 36 questions, *CPAx* Chelsea Critical Care Physical Assessment Tool, *MMSE* Mini- Mental State Examination, *IES-R* Impact of Event Scale- Revised, *BDI-II* Beck Depression Inventory 2, *MCS-12* Mental health Component Summary- 12 questions, *HADS* Hospital Anxiety and Depression Scale, *RBANS* Repeatable Battery for the Assessment of Neuropsychological Scale

^¶^Rose et al., (2021) was the only included study which reported to have done co-production and patient and public involvement work during the eHealth intervention phase

^§^Treatment as Usual control groups underwent usual outpatient clinics

^*^Active control groups were provided education materials and a face-to-face equivalent of the eHealth intervention

### Interventions targeting PICS

There was a wide range of different eHealth interventions and delivery formats. 3 studies investigated telerehabilitation [27, 31, 33], 2 studies investigated telemedicine [26, 35], 2 studies investigated patient monitoring [29, 30], 3 studies investigated virtual reality [36–38], and 1 study investigated a self-directed eHealth intervention [28].

Out of the three domains, eHealth interventions targeted the psychological domain most frequently [26, 28, 33, 35–37], followed by the physical domain [26, 27, 29–31] and the cognitive domain being the least targeted [27, 31, 38]. Only three study teams designed interventions that covered two PICS domains [26, 27, 31]. There were no eHealth interventions that targeted all three PICS domains in tandem. Table 2 summarises the relationship between the intervention delivery format and the domains targeted.

**Table 2:** Summary of targeted PICS domains of each eHealth intervention

Author/Year	Intervention delivery format	Physical	Psychological	Cognitive
Denehy et al. [29]	Wearable sensor	x		
Estrup et al. [30]	Wearable sensor	x		
Jackson et al. [31]	Video conference	x		x
Capin et al. [27]	Video conference	x		x
Balakrishnan et al. [26]	Video conference	x	x	
Park et al. [33]	Video conference		x	
Cox et al. [28]	Application		x	
Rose et al. [35]	Web/application		x	
Vlake et al. [36]	Virtual reality		x	
Vlake et al. [37]	Virtual reality		x	
Wood et al. [38]	Virtual reality			x

### Timing of interventions

Most of the included studies (5/11 studies) chose the early post discharge phase [26–28, 31, 35]. Three studies [29, 30, 36] were conducted in-hospital and 3 studies during the late post-discharge [33, 37, 38].

### eHealth intervention effects on PICS outcomes

#### Outcome measures

There were a variety of outcome measures for each PICS domain. Physical measures include 6MWT [26, 29], TUG [27, 29, 31], CPAx [30], actigraphy step count [29, 30], and 30-s chair stand [27]. Psychological outcome measures included the HADS [37], PHQ [28, 33], GAD-7 [28, 33], BDI-II [36], SF-36 [29], MCS-12 [36], PTSS [28] and IES-R [36, 37]. Cognitive measures included MoCA [27], MMSE [31], RBANS [38]. Studies measuring Health-Related Quality of Life all used the EQ-5D-5L [28, 36, 37].

#### Physical outcomes

The impact of eHealth interventions on physical function was mixed. Whilst Jackson et al. [31] found a significant effect on physical function with a multi-component telerehabilitation, Capin et al. [27] did not find any significant effects on physical function with a tele-physical therapy intervention. A significant improvement in physical function at 3 months post-discharge was significantly correlated with mean daily activity [30]. An absence of chronic disease is a majorly significant (*p* < 0.000) predictor of increased distance walked post-hospital discharge explaining 33.5% of the variance in mean distance walked [29].

#### Psychological and cognitive outcomes

Of the 6 studies that targeted psychological outcomes, 4 studies showed significant reductions in anxiety [33], depression [28, 33, 36], and Post Traumatic Stress Disorder [28, 36]. Only 2 studies showed no effects [26, 37].

Two studies that targeted cognitive outcomes used the same telerehabilitation programmes used in the physical outcomes section [27, 31]. Capin et al. [27] did not find any improvement in cognitive outcomes while Jackson et al. [31] found significant improvement in executive functioning. Wood et al. [38] tested a cognitive screening tool and found less pronounced cognitive impairment 12 months after hospital discharge.

### Secondary outcomes

#### Feasibility

All the included studies which explored feasibility (9 out of 13 studies) demonstrated the feasibility of the various eHealth interventions. Outcome measures used to evaluate feasibility varied. All studies used adherence as an outcome of feasibility. Other outcomes include Attrition [28], safety through reported adverse events [27], VR immersion, and motion sickness [36]. All studies had an adherence rate of more than 70%. One study had 71% adherence [28], 4 studies had > 75% adherence [29–31, 33], 1 study had 83% adherence [27], 1 study had 90% adherence [26], and 2 studies had 100% adherence [36, 37]. A summary of the defined feasibility outcomes and findings is summarised in Additional file 1: Table S3.

#### Acceptability of eHealth interventions

Studies which reported acceptability (5 out of 13) included two qualitative studies [32, 34] and 3 RCTs [26, 27, 37]. Acceptability measures mainly evaluated participant satisfaction and perceptions of the intervention. All studies concluded the intervention to be acceptable. The 3 RCT studies evaluated acceptability using a questionnaire and reported high participant satisfaction.

The two qualitative studies focused on the experiences of a telemedicine intervention and an app-based mood monitoring prototype system [32, 34]. Both studies assessed acceptability through semi-structured interviews and reported barriers and facilitators in intervention uptake.

Most themes considered the sensitivity of mental health and cognitive issues as barriers. Participants from Kovaleva et al. [32] study mentioned that neuropsychological assessments felt ‘embarrassing’ when other clinicians were present in the video call while participants in Parker et al. [34] study thought ‘depression’ was too stigmatising and suggested the term emotions/states as an option.

Usability and perceived usefulness were identified as the main facilitators of the use of eHealth interventions. Facilitators in the acceptability of eHealth interventions included the ease of using the intervention platforms, the convenience, and viewing the platform as a motivator of recovery. A summary of all the acceptability findings can be found in Additional file 1: Table S4.

### Quality assessment and risk of *bias* of included studies

Quality assessments used the MMAT tool [24] with most studies running quantitative randomised controlled trials. Though included RCTs varied in quality, most of the RCT studies were of high quality with 4 of 6 studies scoring 80% [27, 28, 31, 37] and 2 studies were of moderate quality scoring 60% [26, 36]. The main limitations impacting study quality were due to incomplete outcome data and the inability to ‘blind’ participants. There was a greater variance in study quality for non-randomised quantitative studies with 2 high-quality studies scoring 80% [29, 30], 2 studies moderate quality studies scoring 60% [33, 38] and 1 low quality study scoring 20% [35]. The main limitations that impacted the low-quality study were the representativeness of the sample, selection of measures, and incomplete description of intervention as intended. The two qualitative studies were high-quality at 80% [32] and 100% [34]. The detailed rating and scoring of the MMAT tool can be found in Table 3

**Table 3:** Mixed method appraisal tool risk or bias rating scores

Author (Year), Country	1.1	1.2	1.3	1.4	1.5	2.1	2.2	2.3	2.4	2.5	3.1	3.2	3.3	3.4	3.5	Score(%)
Qualitative																
Kovaleva et al. [32]	Y	N	Y	Y	Y											80%
Parker et al. [34]	Y	Y	Y	Y	Y											100%
Quantitative Randomised																
Balakrishnan et al. [26]						Y	Y	N	N	Y						60%
Capin et al. [27]						Y	Y	Y	N	Y						80%
Cox et al. [28]						Y	Y	Y	Y	N						80%
Jackson et al. [31]						Y	Y	Y	Y	N						80%
Vlake et al. [36]						Y	Y	Y	N	N						60%
Vlake et al. [37]						Y	Y	Y	N	Y						80%
Quantitative Non-randomised																
Denehy et al. [29]											Y	Y	Y	N	Y	80%
Estrup et al. [30]											Y	Y	Y	N	Y	80%
Park et al. [33]											Y	N	Y	N	Y	60%
Rose et al. [35]											N	Y	N	U	U	20%
Wood et al. [38]											Y	Y	N	N	Y	60%

*Y* Yes, *N* No, *U* Unknown/Can’t Tell

## Discussion

The main objectives of the study were to systematically assess and explore eHealth’s effectiveness in alleviating PICS impairments, when in the recovery path these are implemented, and the domains being targeted by each intervention. There was a great variety of eHealth interventions with most studies focussing on the physical and psychological domains. Most studies were conducted in the early post-discharge phase and had feasibility as a primary outcome. There is great heterogeneity in the outcome measures used to assess PICS domains, feasibility and acceptability. Nevertheless, findings from the review suggest that eHealth interventions are feasible in a post-critical care setting with further research required in measuring effectiveness.

Though there is variation in the outcome measures used to assess PICS outcomes, the majority of the studies used measures recommended by published core outcome sets (COS). The lack of consistency is due to the different COS available. Remote physiotherapy interventions used a COS focussing on critical care physical rehabilitation [39], while other interventions used a mixture of clinically based COS [40] and COS for clinical research [41]. COS is produced to reduce outcome measure heterogeneity and enable better data synthesis [42]. However, none of the studies reported which outcome sets the measures were selected from. To meet the aims of producing a COS, future studies should report how measures were chosen and identify if a specific COS was used. This will provide consistency in reporting and ease for researchers to compare results across eHealth interventions.

The effects of the eHealth interventions on PICS outcomes were mixed. This is the case for physical and psychological outcomes. The majority of studies targeting psychological outcomes had more interventions reporting positive effects. Vlake et al. [37] did not find significant improvements in psychological outcomes in a late post-discharge sample. However, a prior study conducted by the same authors found an improvement in psychological outcomes in an in-hospital sample that persisted across other follow-up time points [36]. Prior systematic reviews on post-critical care rehabilitation have highlighted the importance of intervention timing [11, 43]. Just as early mobilisation in the critical care ward can alleviate the risk of PICS development [44], there may be an optimal window across the post-critical care recovery path for certain interventions to be effective.

Cognitive outcomes were the least targeted out of the three PICS domains. Studies investigating this outcome observed improvement with multi-component rehabilitation, Jackson et al. [31] attributed significant effects in physical and cognitive outcomes when combining rehabilitation of the two domains together, a result that contrasts with Capin et al. [27] programme which focussed on physical function only. The potential benefits and synergistic effects of performing physical exercise and cognitive training have been documented in other populations [45]. Interrelationships among the three domains are presented through the prevalence of PICS symptom comorbidities. Heesaker et al. [46] observed that mental health and cognitive impairment always occur simultaneously with the other two domains. Marra et al. [47] reported a combination of mental health and cognitive impairment occurring more frequently than other combinations. Kang et al. [48] built on those studies and found that 41.1% of critical care survivors with PICS had symptoms in two or more domains with Physical-Mental symptoms being the most prevalent. With these potential effects, the review found that there has yet to be an intervention that targets these three domains. The incorporation of the cognitive domain is still incipient, and more evidence is required to determine the impact of multi-component interventions.

None of the included studies reported on a theory of behaviour change and only one study [35] reported evidence of co-producing the intervention. Recent guidelines from the Medical Research Council recommend complex health interventions to be co-produced and underpinned by the behavioural theory of change as it increases the effectiveness of behaviour change [49–51]. There is a possibility that behavioural theories have been implied and not discussed explicitly. Goal setting was used in the digital pathway intervention by Rose et al., [35], app-based Mindfulness [28] and tele-psychotherapy [33] rely on the mechanisms of change brought by the therapeutic approaches. Nevertheless, explicit reporting of theories used as well as evidence of co-production is integral in evaluating complex health interventions.

Most studies point to the feasibility of implementing eHealth interventions. With regards to acceptability, studies that assessed it deemed the eHealth interventions feasible. The implementation of eHealth interventions into day-to-day clinical practice has been challenging [52]. The decision to adopt an eHealth intervention requires careful management of both patient and staff expectations [53]. Clinicians and hospital staff need to believe that the intervention can improve care and efficiency. They need to be on board, involved, and receive consistent support during the adoption [54]. The success of eHealth implementation is also determined by patient engagement and uptake. This is especially challenging in older patient populations like critical care survivors. The themes of usability and perceived usefulness highlighted in this review were in line with older patients with chronic conditions [55], older patients with cancer [56], and the general older population [56, 57]. Critical care survivors were more likely to adhere to eHealth interventions when they are easy to use, convenient and perceived as a motivator towards recovery. The continuous contact between patients and the clinical team through telemedicine visits supported the perceptions of care continuance, thus increasing the perceived usefulness and adherence to eHealth interventions. Despite the alignment with research on senior populations, acceptability was only assessed by 5 out of 13 studies which limits the generalisability of findings in a post-critical care population. Further research is needed to address the specific barriers and facilitators for eHealth uptake and engagement in this population.

### Study limitations

One limitation of this review is the infancy of the current research area. The primary objective of studies included in the review was to assess the feasibility of the intervention resulting in underpowered studies with small samples. The effects of eHealth on each PICS domain are preliminary in nature. Nevertheless, the summarised evidence paints a promising picture of the development of eHealth interventions in this population. Future studies need to focus on larger-scale RCTs which will provide more insight into intervention effectiveness. The authors of the ICU-VR intervention have progressed to a larger RCT trial [59] in hope of generating more robust effects of the intervention on PICS outcomes. Other eHealth trials are also underway in this post-critical care phase of recovery [60–62]. Thus, whilst eHealth interventions can be concluded to be feasible, conclusions on effectiveness are premature at this point.

Even though no restriction was imposed on the language and country of article publication, the language used in the search strategy undoubtedly constrained its results. We acknowledge that if the search terms included other languages, other articles could be deemed eligible. This review adhered closely to the PICO framework [22] and search strings were systematically piloted in preliminary searches. The review attempted to be as broad as possible regarding the search strategy and the databases selected. Future research may also benefit from the inclusion of Medical Subject Headings (MeSH) terms to further expand the search.

## Conclusions

eHealth research and development in post-critical care rehabilitation is still early in its infancy with most studies focusing on feasibility. Based on the review findings, preliminary feasibility results are promising with research progressing to larger scale studies to derive more robust conclusions on effectiveness. Future research should be prioritised towards acceptability, targeting the cognitive domain, and exploring the effects of interventions targeting all 3 domains. eHealth is one vital solution in providing access, continuity, and sustainable care in the post-critical care setting.

## Supplementary Information

**Additional file 1. Supplementary Material S1**. Database Search Strategies. **Supplementary Material S2**. Quality Assessment Criteria of the Mixed Method Appraisal Tool based on Study Design. **Table S3**. Feasibility outcomes and findings of eHealth interventions. **Table S4**. Acceptability Outcomes and findings of eHealth interventions

## Data Availability

Supplementary materials are provided and can be assessed online.

## Abbreviations

ICU: Intensive Care Unit
PICS: Post Intensive Care Syndrome
HRQoL: Health Related Quality of Life
eHealth: Electronic Health
PRISMA: Preferred Reporting Items for Systematic Reviews and Meta-Analyses
PROSPERO: Prospective Register of Systematic Review databases
MMAT: Mixed Methods Appraisal Tool
RCT: Randomised Controlled Trial
TAU: Treatment as usual
Medical Subject Headings: MeSH
