# Supplementary material for: Exploring the effectiveness of eHealth interventions in treating Post Intensive Care Syndrome (PICS) outcomes: a systematic review
Source: Crit Care. 2024 Sep 27;28:317. doi: 10.1186/s13054-024-05089-6 (PMC11428403; doi:10.1186/s13054-024-05089-6)
Supplement: Supplementary file 1 — Additional file 1. Supplementary Material S1. Database Search Strategies. Supplementary Material S2. Quality Assessment Criteria of the Mixed Method Appraisal Tool based on Study Design. Table S3. Feasibility outcomes and findings of eHealth interventions. Table S4. Acceptability Outcomes and findings of eHealth interventions [file 13054_2024_5089_MOESM1_ESM.pdf]

Supplementary Material

Exploring the effectiveness of eHealth interventions in treating Post Intensive Care Syndrome (PICS) outcomes: a systematic review.

Daniel Lai Jie, Zhao Liu, Elaine Johnston, Lisa Dikomitis, Teresa D'Oliveira, Sukhi Shergill

Supplementary Material S1. Database Search Strategies

Supplementary Material S2: Quality Assessment Criteria of the Mixed Method Appraisal Tool based on Study Design

Table S3. Feasibility outcomes and findings of eHealth interventions

Table S4. Acceptability Outcomes and findings of eHealth interventions

Supplementary Material S1. Database Search Strategies

Medline, Embase, CINAHL

The search string was (eHealth OR e-health OR mhealth OR tele-health OR telemedicine OR remote monitoring OR remote patient monitoring OR Wearable monitors OR physiological monitoring OR Virtual Reality) AND (Intensive Care Unit OR ICU OR Critical care OR critical care survivor* OR post intensive care discharge OR post-ICU OR Post Intensive Care Syndrome OR PICS) AND (Physical function OR ICU-acquired weakness OR Psychological outcomes OR Depression OR Anxiety OR PTSD OR Cognitive Outcomes OR Memory OR Attention OR executive function).

Scopus

{eHealth} OR {e-health} OR {mhealth} OR {tele-health} OR {telemedicine} OR {remote monitoring} OR {remote patient monitoring} OR {Wearable monitors} OR {physiological monitoring} OR {Virtual Reality}
AND
{Intensive Care Unit} OR {ICU} OR {Critical care} OR {critical care survivor} OR {post intensive care discharge} OR {post-ICU} OR {Post Intensive Care Syndrome} OR {PICS}
AND
{Physical function} OR {ICU-acquired weakness} OR {Psychological outcomes} OR {Depression} OR {Anxiety} OR {PTSD} OR {Cognitive Outcomes} OR {Memory} OR {Attention} OR {Executive function}

PsycINFO
(TITLE-ABS-KEY({eHealth} OR {e-health} OR {mhealth} OR {tele-health} OR {telemedicine} OR {remote monitoring} OR {remote patient monitoring} OR {Wearable monitors} OR {physiological monitoring} OR {Virtual Reality})
AND
TITLE-ABS-KEY({Intensive Care Unit} OR {ICU} OR {Critical care} OR {critical care survivor} OR {post intensive care discharge} OR {post-ICU} OR {Post Intensive Care Syndrome} OR {PICS})
AND
TITLE-ABS-KEY({Physical function} OR {ICU-acquired weakness} OR {Psychological outcomes} OR {Depression} OR {Anxiety} OR {PTSD} OR {Cognitive Outcomes} OR {Memory} OR {Attention} OR {Executive function}))

Supplementary Material S2: Quality Assessment Criteria of the Mixed Method Appraisal Tool based on Study Design

1. Qualitative

1.1. Is the qualitative approach appropriate to answer the research question?

1.2. Are the qualitative data collection methods adequate to address the research question?

1.3. Are the findings adequately derived from the data?
1.4. Is the interpretation of results sufficiently substantiated by data?
1.5. Is there coherence between qualitative data sources, collection, analysis and interpretation?
2. Quantitative randomized controlled trials
2.1. Is randomization appropriately performed?
2.2. Are the groups comparable at baseline?
2.3. Are there complete outcome data?
2.4. Are outcome assessors blinded to the intervention provided?
2.5 Did the participants adhere to the assigned intervention?
3. Quantitative non-randomized
3.1. Are the participants representative of the target population?
3.2. Are measurements appropriate regarding both the outcome and intervention (or exposure)?
3.3. Are there complete outcome data?
3.4. Are the confounders accounted for in the design and analysis?
3.5. During the study period, is the intervention administered (or exposure occurred) as intended?

Table S3. Feasibility outcomes and findings of eHealth interventions

Author	Defined Feasibility Outcome	Attrition	Adherence measure	Adherence	Reasons for withdrawal	Author's conclusions
Balakrishnan et al., 2023[26]	Adherence	10%	100% of components	90%	Lost to follow-up	Telemedicine clinic shown to be a feasible and acceptable model of follow-up on Covid-19 and ARDS survivors.

Capin et al., 2022[27]	• Session adherence (% of 12 sessions attended)<br>• Safety (no. Of adverse events)	17%	≥ 75% of all sessions	83%	Lost to follow-up	Program concluded to be feasible and safe (no reported adverse event) to conduct a multi component telerehabilitation programme.
Cox et al., 2019[28]	- Attrition<br>- Session adherence (% of 4 sessions)	29%	100% of sessions	71%	Lost to follow-up<br><br>Medical issues<br><br>Work	All prespecified criteria met except attrition.<br>Feasibility was concluded as majority of attrition occurred before the start of intervention
Denehy et al.,2012 [29]	- Adherence (% of 7-day actigraphy)	23%	57.1 % (≥ 4 days of the week)	77%	Incomplete actigraphy data	Based on adherence rates of actigrahy monitoring, monitoring patients in ICU was deemed feasible
Estrup et al., 2019[30]	- Adherence (% of 7-day actigraphy)	20.6%	71.4% (≥ 5 days of the week)	79.4%	Declined visit<br><br>Died<br><br>Lost to follow-up	Using actigraphy to monitor patients in ICU was deemed feasible based on adherence.
Jackson et al., 2012[31]	-Adherence (% of sessions)	23.1%	100% of sessions	76.9%	Work<br><br>Medical issues	Feasibility established to conduct telerehabilitation program in post-icu population. There was a 76.9% adherence to intervention
Park et al., 2023[33]	-Adherence (% of 14 sessions)	22%	≥50% sessions	77.8%	Lost to follow-up<br><br>Hospital readmission	Feasibility established in using telepsychotherapy
Vlake et al., 2021[36]	-Motion sickness (simulation sickness questionnaire)<br>-VR immersion (group presence questionnaire)<br>-Safety (adverse events)<br>-Follow-up rates	8%	Follow-up rates after 30 days	100%<br>96% 7-day follow up<br>92% 30-day follow up	Death<br><br>Lost to follow -up	Authors concluded intervention to be feasible and safe from the low motion sickness scores, high VR immersion scores, and no reported adverse events
Vlake et al., 2022[37]	Adherence to intervention	0%	Follow-up rates after 4-months	100%<br>100% 3 month follow up	None reported	Authors concluded feasibility from adherence rates of attending follow up assessments after 4 months

				100% 4-month follow-up		

ARDS Acute respiratory distress Syndrome, *ICU* Intensive Care Unit, *VR* Virtual Reality

Table S4. Acceptability Outcomes and findings of eHealth interventions

Author, Year	Acceptability Measure	Main Findings	Acceptability	
			Facilitators	Barriers

Balakrishnan et al., 2023[26]	Semi-structured interviews	• Themes identified through content analysis demonstrated participant's acceptability to telemedicine clinic intervention<br>• Participants found the appointment and provided devices acceptable and useful. 75% of participants continued using provided pulse oximeter and BP monitor after study's conclusion<br>• Telemedicine follow-up was found to be reassuring	Not reported	Not reported
Capin et al., 2022[27]	Not assessed			
Cox et al., 2019[28]	Client Satisfaction Questionnaire	• Client satisfaction questionnaire acceptability measure, which scored out of 36, exceeded benchmark scores (M= 27.6, SD = 3.8).	Not reported	Not reported
Denehy et al., 2012[29]	Not assessed			
Estrup et al., 2019[30]	Not assessed			
Jackson et al., 2012[31]	Not assessed			
Kovaleva et al., 2023[32]	Semi-structured interviews	• All participants found telemedicine visit acceptable<br>• Intervention organisation and delivery<br>-Technology was easy to use	• Professionalism demonstrated by the multi-disciplinary team<br>• Usability of the technology<br>• Convenience	• Completing neuropsychological assessment in front of other clinicians felt 'embarrassing'.<br>• Some participants were uncomfortable with having mental

		• Intervention substance<br>-rehabilitation clinic was very detailed in covering topics around post-hospital recovery<br>- reassured about mental health status<br>• Ways to improve intervention<br>• Use computer as main mode of eHealth service delivery<br>• Increase visit frequency<br>• Schedule visit sooner<br>• Match visit schedules with individual recovery trajectories		health status evaluated
Park et al., 2023[33]	Not assessed			
Parker et al., 2020[34]	Question rating app helpfulness And semi-structured interviews	• 80% stated application was easy to use<br>• 37% stated it would be helpful to have an application demonstration<br>• Median score for app 'helpfulness' was the maximum score of 5, IQR [4.24-5.00]	• Seeing the application as a motivator of recovery<br>• Setting achievable goals with the application to encourage survivors to engage<br>• Adding reward systems (i.e inspirational quotes, emoticons, coupons for online game)	• Stigma of depression. Participants wanted the option to track a variety of emotions/states<br>• Access to smartphones or tablets
Rose et al., 2021[35]	Not assessed			
Vlake et al.,2021 [36]	Not assessed			
Vlake et al., 2022[37]	Quality and satisfaction questionnaire	• ICU-VR group rated satisfaction of ICU after care	Not Reported	Not Reported

		significantly higher (p =.002)<br>• ICU-VR added satisfaction of ICU care according to 62% of patients<br>• 100% of ICU-VR group would recommend the VR intervention to other patients		
Wood et al, 2018[38]	Not assessed			

BP Blood Pressure, *M* Mean, *SD* Standard Deviation, *IQR* Inter Quartile Range, *ICU* Intensive Care Unit, *VR* Virtual Reality
